# Global burden of neuroendocrine tumors and changing incidence in Kentucky

**DOI:** 10.18632/oncotarget.24983

**Published:** 2018-04-10

**Authors:** Aman Chauhan, Qian Yu, Neha Ray, Zainab Farooqui, Bin Huang, Eric B. Durbin, Thomas Tucker, Mark Evers, Susanne Arnold, Lowell B. Anthony

**Affiliations:** ^1^ Markey Cancer Center, University of Kentucky, Lexington, KY, USA; ^2^ Department of Internal Medicine, University of Kentucky, Lexington, KY, USA; ^3^ University of Cincinnati School of Medicine, Cincinnati, Ohio, USA; ^4^ Kentucky College of Osteopathic Medicine, Pikeville, KY, USA; ^5^ Department of Biostatistics, University of Kentucky, Lexington, KY, USA; ^6^ Kentucky Cancer Registry, Lexington, KY, USA; ^7^ Department of Surgery University of Kentucky, Lexington, KY, USA

**Keywords:** neuroendocrine tumor incidence, kentucky cancer registry, SEER

## Abstract

**Background:**

Neuroendocrine tumors (NETs) have a low incidence but relatively high prevalence. Over the last three decades, the incidence of NETs has risen 6-fold in the United States. We conducted an observational study to compare the incidence of NETs reported to the Kentucky Cancer Registry (KCR) versus that reported to Surveillance, Epidemiology, and End Results Program (SEER). We also provide a systematic review of the state of neuroendocrine tumors worldwide, and compare the available global and local published data.

**Methods:**

KCR and SEER databases were queried for NET cases between 1995 and 2015. A detailed literature review of epidemiological data for various nations worldwide summarize epidemiological data from various countries.

**Results:**

KCR recorded 6179 individuals with newly diagnosed NETs between 1995 and 2015. Between 1995-2012, the incidence of NETs in KCR increased from 3.1 to 7.1 per 100,000 cases, while it increased from 3.96 to 6.61 in the SEER database. The incidence rates in both KCR and SEER databases were linear. 90.57% were Caucasians with 54.74% females. 27.67% of the Kentucky population was from the Appalachian region. Patients aged 50-64 years had the highest prevalence (38%). Lung NET (30.60%) formed the bulk of cases, followed by small intestine (16.82%), rectum/anus (11.35%) and colon (9.71%).

**Conclusions:**

NETs incidence between 1995 and 2015 show a linear increase in both KCR and SEER databases. Because of this increased incidence it is imperative for community oncologists to familiarize themselves with this entity, which until recently was under-studied and with few viable treatment options.

## INTRODUCTION

Neuroendocrine tumors are frequently grouped with other rare malignancies, which limits drug development and clinical trial expansion for this important oncologic entity. Fortunately, the last 15 years have seen rapid changes in awareness and perception of general oncologists toward neuroendocrine tumors. As a result, there is not only an expansion of evidence-based standard of care data for the treatment of these patients, but future therapeutic drug developmental avenues also look promising. Robust epidemiological surveillance of this population helps us recognize the quantum of disease burden and unique patterns of disease presentation, which are key in the use of effective screening, targeted diagnostics, and treatments.

We performed a retrospective analysis of the Kentucky Cancer registry (KCR) and the Surveillance, Epidemiology, and End Results Program (SEER) databases between 1995 and 2015. Studies outside of the U.S. were also analyzed in terms of incidence, anatomical sites, grade, and survival. The aim of the study is to analyze epidemiologic and demographic data of newly diagnosed neuroendocrine tumors in Kentucky and compare this information with currently available global data.

## RESULTS

We identified 6179 individuals with newly diagnosed NET between 2005 and 2015. We observed a steady rise in annual incidence of NET from 119 new cases in 1995 to 528 cases in 2015 (Table 1). After accounting for Kentucky’s population increase (from 4.183 million to 4.425 million during those years), we determined that the corresponding incidence rate increased from 3.1 to 10.3 per 100,000 population (Table 1). Of our study patients, 90.57% were Caucasian with 54.74% females. Patients aged 50-64 years had the highest prevalence (38%). Geographically, 27.67% of our Kentucky population was from the Appalachian region. Lung NET (30.60%) formed the bulk of cases, followed by small intestine (16.82%), rectum/anus (11.35%) and colon (9.71%) (Table 2A). 46.92% of females presented with localized disease as compared to 36.07% males. 26.45 % of African Americans NET patients were aged between 20-49 years as compared to 18.22% of Caucasian patients. Lung NET was highest among smokers, whereas small bowel NET was highest among nonsmokers.

**Table 1:** KCR and SEER NETs cases, 2005-2015

Year	KCR (age adjusted incidence)	SEER (age adjusted incidence)	KCR (Cases)	SEER (Cases)
**1995**	3.1	3.96	119	1299
**1996**	3.6	4.12	138	1372
**1997**	3.6	4.29	141	1456
**1998**	4.7	4.73	188	1631
**1999**	5	4.76	204	1671
**2000**	5.3	4.9	218	3613
**2001**	5.2	4.67	214	3510
**2002**	5.5	5.14	232	3947
**2003**	5.2	5.29	226	4123
**2004**	5.9	5.44	257	4335
**2005**	5.8	5.56	257	4378
**2006**	6.3	5.94	281	4880
**2007**	7.4	6.16	342	5196
**2008**	7.4	6.27	351	5401
**2009**	7.2	6.38	346	5628
**2010**	8.2	6.57	405	5913
**2011**	7.1	6.61	351	6042
**2012**	8.4		414	
**2013**	8.6		447	
**2014**	10.4		520	
**2015**	10.3		528	

**Table 2A:** Site-specific prevalence of neuroendocrine tumors in Kentucky

Site	Prevalence (%)
Lung	30.6
Small Intestine	16.82
Rectum/Anus	11.35
Colon	9.71
Pancreas	5.5
Others	12.91
Unknown	7.61

The majority of cases (52%) were low grade, with 22% high grade, 3% intermediate grade, and the rest unknown (Table 2B). Grading of tumors is based on the globally-accepted WHO criteria, which considers Ki 67 <2% low grade, 2-20% intermediate grade, >20% high grade [1]. This grading system might be modified in the future based on recent data from Tang et al. [2] It should be noted that roughly 25% of newly diagnosed NET patients are histologically placed at either G2 or G3. These patients have a distinct clinicopathological course as compared to low grade (G1) NETs.

**Table 2B:** Grade specific distribution of NETs in Kentucky

WHO 2010 Grading	%
G1	52
G2	3
G3	22
Unknown	23

Unlike most other malignancies, NETs are usually diagnosed at an early stage. In our study population, 42% had local disease at the time of diagnosis, 22% had loco-regional disease, 22% presented with distant metastatic disease, and the other 14% had an unknown status. Except for those with local disease, where 25% were females as compared to 16% males, the loco-regional and metastatic subgroups did not show any gender discrepancy. Staging has a profound impact on the choice of treatment, with early stage being curative with surgical resection alone, especially for low grade histology. As expected, 52% of our study population underwent surgery alone as the treatment of choice.

As far as age stratification is concerned, peak incidence was found in 50-64 year age group (38%), followed by 65-74 (25%), 20-49 (19%) and 75+ (18%). These data are in agreement with most found in the published literature.

Lastly, 27% of our study population reside in the Appalachian region. Multivariate analysis of the Appalachian population did not show significant aberration in patterns compared with the non-Appalachian population with regard to any of the previously discussed study variables.

## DISCUSSION

Derived from the neural crest cells during development, NETs can arise from various primary sites in the body. Neuroendocrine tumors are commonly found in the gastrointestinal tract but it is not unusual to find neuroendocrine tumors in lungs, pancreas, thyroid, gonads, and other locations [3–5]. Besides site of origin, NETs can also be subdivided between secretory or non-secretory subgroups based on production of hormones or into high grade, intermediate grade or low-grade tumors based on the pathology. There is substantial heterogeneity among all these subgroups in terms of natural history of disease progression, response to therapeutic agents, and overall survival. Despite their low incidence rate, all NETs harbor the potential for malignancy, rendering the term “carcinoid tumor,” adopted by previous researchers, inaccurate. In addition to their misnomer, the diagnosis criteria and classifications of NETs have been under debate since their discovery at the beginning of the last century. JACC, WHO, and ENETS criteria standards differ among each other, and the criteria for NETs from various sites may also vary within the same guideline. Along with the lack of population-based NET registries across the world in general, these variances have prevented us from elucidating the worldwide epidemiology of NETs over time. Rather, a multitude of studies emerged to assess the prognostic value of each classification scheme. In this systematic review, we examined NET cohort studies by country, focusing on the profile of NET composition and incidences, to gain a worldwide perspective of NET epidemiology.

### Primary sites

Consistent with the SEER database, the most common NET sites in KCR database were lung, small intestine, and rectum-anus (Table 2A) [6]. A nation-wide study from Netherland also reported pulmonary NET being the most prevalent. In contrast, two studies from Denmark and Sweden reported small intestine NETs to be most prevalent [7, 8]. If only gastroenteropancreatic neuroendocrine tumors (GEP-NETs) were considered, studies in Argentina, France, and Norway reported the small intestine as being the most common anatomical location [9–11]. Table 3 summaries the three most prevalent NET sites globally. Appendix NET was prevalent among Western countries in general, which could be explained by accidental findings during appendectomy. The survival rates of appendix NET were also the most optimal, which might also result from early detections (Table 4). In contrast, rectal NET was particularly common in Asian populations [12–15]. Whether these distinctions resulted from genetic variations needs further research.

**Table 3:** Top 3 primary sites of NETs, reported by country

Country	Authors	Years	Sample size	1^st^ Most Common Primary Site	2^nd^ Most Common Primary Site	3^rd^ Most Common Primary Site
Argentina	[9]	NA	532 (GEP-NET only)	Small Intestine (26.9%)^1^	Pancreas (25.2%)	Colon-Rectum-Anus (12.4%)
Austria (prospective study)	[22]	2004-2005	265	Stomach (23%)	Appendix (21%)	Small Intestine (15%)
Brazil	[31]	1997-2009	773 (GEP-NET only)	Stomach (24.5%)	Small Intestine (20.8%)	Rectum (20.5%)
Canada	[23]	1994-2009	5619	Pancreas (25%)	Colon (22.8%)	Small Intestine (21.6%)
Canada	[25]	1990-2005	530	Small Intestine (55.3%)	Colon (18.3%)	Appendix (17.9%)
China	[13]	2009-2013	248 (GEP-NET only)	Rectum (30.6%)	Pancreas (23.4%)	Gastric (13.3%)
China	[32]	1991-2013	130 (NET with liver metastasis only)	Pancreas (65.4%)	Stomach (10.8%)	Small Intestine (5.4%)
China	[33]	2011-2016	440 (GEP-NET only)	Stomach (24.3%)	Rectum (24.1%)	Pancreas (20.5%)
China (Hongkong)	[34]	1994-2013	126 (GEP-NET only)	Pancreas (34.9%)	Rectum (33.3%)	Stomach (8.7%)
Denmark	[7]	1978-1989	1029	Small Intestine (29.4%)	Appendix (17.9%)	Pulmonary (16.8%)
France	[10]	2001-2002	668 (GEP-NET only)	Small Bowel and Colon (43%)	Pancreas (32%)	Gastric (5%)
Germany	[35]	2004-2007	1263	Pancreas (31%)	Small Intestines (22%)	Colon and Rectum (11.4%)
Italy	[36]	2004-2007	1203	Pancreas (31%)	Lung (29%)	Ileum, Cecum, Colon, Rectum (13%)
Korea	[14]	2000-2009	4951 (GEP-NET only)	Rectum (48%)	Stomach (14.6%),	Colon (7.9%)
Lebanon	[37]	2001-2012	89 (GEP-NET)	Pancreas (24.7%)	Stomach (20.8%)	Duodenum (18.2%)
Mexico	[38]	NA	495 (GEP-NET only)	Pancreas (33.27%)	Stomach (28.02%)	NA
Norway	[24]	1993-2004	2030	Small Intestine (25.5%)	Lung and Bronchus (21%)	Colon (8%)
Norway	[11]	2003-2013	204 (GEP-NET only)	Small Intestines (29.4%)	Appendix (23.5%)	Pancreas (16.2%)
Netherlands	[21]	2001-2010	24759	Pulmonary (72.7%)	Appendix (3.9%)	Small Intestine (3.7%)
Spain	[39]	2001-2008	907	Pancreas (34%)	Jejunum-Ileum (15.6%)	Appendix (9.4%)
Sweden	[8]	1987-2012	7334	Small Intestine (32%)	Appendix (23%)	Lung (15%)
Taiwan	[15]	1996-2008	2187	Rectum (25.4%)	Lung/Bronchus (20%)	Stomach (7.4%)
United States (by incidence)	[6]	2000-2012	64971	Lung (1.49%)	Small Intestine (1.05%)	Rectum (1.04%)

^1^Values in parentheses are % of study cohort.

**Table 4:** Five-year overall survival (OS) rate of NET by primary site (%)

Country	Authors	Rectum	Lung	Stomach	Pancreas	Colon	Small Intestine	Appendix
Canada	[25]	100		70		73	70	84
Germany	[19]	50		53	52	48	68	86
Norway	[24]	74	54	45	43	41	59	74
Spain	[39]	64.1		61.4	78.1	65.1	Duodenum (89.3), Jejunum-Ileum (83.0)	100
Taiwan	[21]	80.9	33.9	46.4	30.2	48.1	47.9	75.7
U.S. (Distant stage G1/G2 only; diagnosed from 2000-2012)	[6]	28	32	32	50	29	69	
U.S. (reported by median OS time in years)	[6]	24.6	5.5		3.6			>30

### Grading and survival

From a global perspective, few countries reported grading data according to Ki-67 and/or mitotic counts due to the continuously evolving grading systems. The percentages of each grade varied among studies. Although a nation-wide, multicenter study in Korea reported a high G1 percentage of 92.3%, a regional study in China indicated that 73.9% of all graded cases were G3. Nevertheless, a consistent impression among all studies was that lower grade NETs demonstrated a better outcome in terms of survival. Tables 4 and 5 summarize global NET 5-year overall survival by site and by grade. Grade 1, Grade 2, and Grade 3 in our Kentucky population were 67.5%, 28.6%, and 3.9%, respectively (Table 2B). Table 6 summarizes global prevalence of NETs by grade. Our data underscores the fact that roughly 25% of newly diagnosed NET patients in Kentucky present with either G2 or G3 histology. This is a significant number considering these intermediate grade and high grade NETs are often aggressive and have very limited treatment options.

**Table 5:** Five-year OS rate of NET by grade (%)

Country	Authors	G1	G2	G3
Argentina(GEP-NET only; values were estimated from graph)	[9]	>75	50-75	<25
China(NET with liver metastasis only)	[32]	55.658.(surgical resected patients); 14.3 (non-surgically resected patients)	35.3 (surgical resected patients); 0 (non-surgically resected patients)	28.6 (surgical resected patients); 0 (non-surgically resected patients)
Korea (GEP-NET only)	[14]	94.2	70.38	42.96
Netherlands (based on the data from 2001-2010)	[21]	80	63	20 (G3-LCNEC), 6 (G3-SCNEC)
Spain (GEP-NET only)	[39]	83.3	77.1	43.5
U.S. (reported by median OS time)	[6]	16.2 years	8.3 years	10 months (including G4)

**Table 6:** Percentages of NET subtypes categorized according to WHO2010 criteria, reported by country

Country	Author	G1	G2	G3	Others (if applicable)
Argentina (GEP-NET only)	[9]	29.5%	35.9%	9.0%	No data (25.6%)
Brazil	[31]	73.2%	10.5%	16.3%	
China	[13]	3.1%	18.0%	73.9%	MANEC (9.1%)
China	[32]	27.3%	51.5%	21.2%	
China	[33]	29.5%	27.3%	43.2%	
China (Hong Kong)	[34]	87.3%	NA	NA	
Germany	[35]	40.4%	51.1%	18.4%	
Korea	[14]	92.31%	4.85%	2.84%	
Lebanon	[37]	56.2%	11.2%,	20.2%,	MANEC (12.4%)
Mexico	[38]	64%	13%	23%	
Netherlands	[21]	17%	1%	G3-LCNEC (7%) G3-SCNEC (75%)	
Norway	[11]	53.4%	24.0%	19.6%	
Spain	[39]	44.4%	37.8%	17.8%	
U.S.	[6]	51.0%	16.4%	32.5% (including G4)	

### Incidence

**Figure 1:**
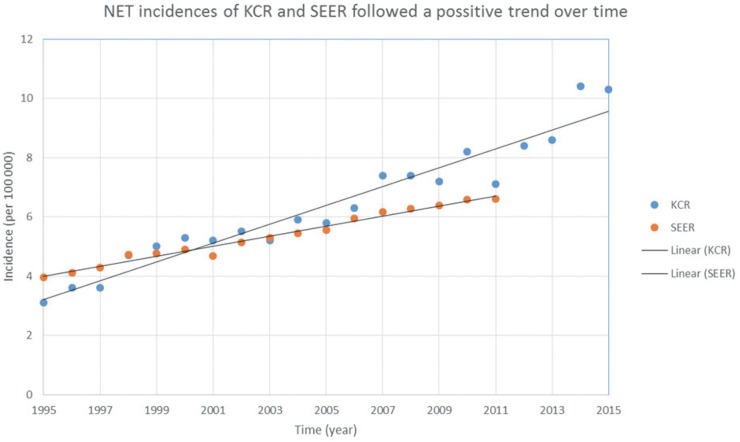
NET incidence according to KCK (blue/series 1) and SEER (red/series 2) [6]

Unlike most malignancies, NET incidence has shown a constant incline. The cause of this increase is not entirely clear but experts have attributed the pattern to an increased awareness and improved diagnostics. The hike in incidence of this otherwise rare tumor has alerted public health stakeholders, which has resulted in establishment of specialized NET registries worldwide [16, 17]. A seminal paper from Yao et al. showed a 5-fold increase in incidence of NET from 1973 to 2004 [3]. Incidence of NET per Yao’s report is estimated to be 5.25 cases per 100,000 population. Similar observations were made by Tsikitis et al. in the GI NET subgroup [18]. Per their report, overall incidence of GI NETs increased in all sites except for appendix tumors. In Germany, the age-standardized incidence increased from 2.7 to 3.4 fold from 1976 to 2006 [19]. In Italy, the incidence increased from 0.7 to 5.3 between 1976 and 2010 [20]. Based on recently published papers, increasing trends were also observed in China Japan, Korea, Norway, Netherlands, and Taiwan [11-15, 21]. Outside of the United States, incidence rates ranged from 1.41 to 5.86 based on studies published after 2000 [13, 15, 19, 20, 22-25]. Based on our analysis, the incidence of NETs reported in Kentucky according to the KCR database increased from 3.1 (1995) to 10.3 (2015) per 100,000 cases. In contrast, according to SEER data, the incidence increased from 3.9 to 6.61 between 1995 and 2012 (Figure 1).

### Limitations

Since the beginning of the last century, the nomenclature, diagnostic criteria and classification of NETs have been evolving. “Carcinoid tumor”, a previous term adopted by several early studies, was inaccurate because all NETs harbor the potential for malignancy [7, 26–30]. Older versions of the International Classification of Disease for Oncology excluded benign NETs. The WHO has changed grading criteria over time. All of these inconsistencies prevent achieving a clear comparison of different epidemiology studies overtime. Most countries do not have a nation-wide, population based NET registries. Single-center or regional studies cannot assess national incidence and prevalence convincingly.

## MATERIALS AND METHODS

KCR is a part of NCI’s SEER program and has been collecting population-based cancer data since 1986. We sought Institutional Review Board approval prior to commencement of our study, then proceeded to review the KCR and SEER databases for all newly diagnosed neuroendocrine tumors in Kentucky between 1995 and 2015. Incidence data were adjusted for population. Multivariate analysis was performed using SPSS to analyze various demographic and disease-specific study variables. Tumors were characterized by primary sites. Grading was based on the globally-accepted WHO criteria, which considers Ki 67 <2% low grade (G1), 2-20% intermediate grade (G2), >20% high grade (G3) [1].

Studies outside of the United States (U.S.) were reviewed according to search results from PubMed and Google Scholar. Examples of key words included: “neuroendocrine+tumor”; “neuroendocrine+neoplasm”; “neuroendocrine+prevalence”; “neuroendocrine+incidence”;” neuroendocrine+burden”; “neuroendocrine+epidemiology”; “carcinoid+tumor”; “carcinoid+incidence. Studies were sorted into tables according to authors, date, sample size, primary sites of tumor, grading, survival. Re-calculations were performed in some cases based on data from original studies in order to exclude unknown type and to obtain percentages of each tumor type and grade.

## CONCLUSIONS

NETs incidence between 1995 and 2015 showed a linear increase in both KCR and SEER databases. NET incidence has undoubtedly increased over the past two decades. It is imperative for community oncologists to familiarize themselves with this entity, which until recently was under-studied and without many viable treatment options.
